# Index medicus for the Eastern Mediterranean region

**DOI:** 10.1186/1742-7622-5-14

**Published:** 2008-09-30

**Authors:** Najeeb Al-Shorbaji

**Affiliations:** 1 Regional Office for the Eastern Mediterranean, World Health Organization, P.O. Box 7608, Cairo (11371), Egypt

## Abstract

The study provides the rationale, history and current status of the Index Medicus for the World Health Organization Eastern Mediterranean Region. The Index is unique in combining the geographic coverage of peer-reviewed health and biomedical journals (408 titles) from the 22 countries of the Region. Compiling and publishing the Index coupled with a document delivery service is an integral part of the WHO Regional Office's knowledge management and sharing programme. In this paper, bibliometric indicators are presented to demonstrate the distribution of journals, articles, languages, subjects and authors as well as availability in printed and electronic formats. Two countries in the Region (Egypt and Pakistan) contribute over 50% of the articles in the Index. About 90% of the articles are published in English. Epidemiology articles represent 8% of the entire Index. 15% of the journals in the Index are also indexed in MEDLINE, while 7% are indexed in EMBASE. Future developments of the Index will include covering more journals and adding other types of health and biomedical literature, including reports, theses, books and current research. The challenges and lessons learnt are discussed.

## Background

Indexing and abstracting services are multipurpose tools. They represent an inventory or a record of literature published according to a certain set of criteria such as subject, geographic coverage or authorship. They form the basis for the development of networks of researchers and authors, and they are the road map to lead information users as to what, where, when a document was published and by whom. An ***index ***is a systematic arrangement of entries designed to enable users to locate information in a document [1]. Indexes are the starting point for accessing information and making it available through document delivery services and online access. From the point of view of the WHO Regional Office for the Eastern Mediterranean (EMRO), the Index Medicus for the Eastern Mediterranean Region (IMEMR) has been maintained to achieve all the above purposes and beyond. Publishing in science and technology including health and biomedical sciences represents a small portion of most of the national information production in the Region, which has resulted in weak indexing and abstracting services. National indexing and abstracting services do not exist in most of the EMR member states. The Index was initiated to fill the gap of the near absence of health and biomedical journals published in the Region from international indexing and abstracting services. Other WHO Regional Offices have initiated similar activities to fulfil similar needs in member states of these regions. The ultimate goal of these indexes is to create a global index medicus that can bridge the gap resulting from lack of indexing of "third world" biomedical literature in MEDLINE and other international systems. This article provides a description and analysis of the IMEMR and how it maps health and biomedical literature in the Region, and acts as a knowledge diffusion mechanism.

Facilitating access to health information is within the WHO constitution. It is stated that " [the] extension to all peoples of the benefits of medical, psychological and related knowledge is essential to the fullest attainment of health" [2]. In implementing this and in response to country needs, the Regional Committee for the Eastern Mediterranean issued a resolution on the Regional Medical Library in which it recommended that "*the Regional Director takes further steps for the consolidation of the resources and services of the WHO Regional Medical Library and the development of a viable Network for biomedical information" *[3]. The unit in charge of health information management interpreted this recommendation by developing of a series of projects and activities, all of which is leading to more health information exchange and dissemination in the Region. Details of these activities and services are on the Regional Office's website [4] as part of the Virtual Health Sciences Library activities.

## History of the IMEMR

One major project in the Virtual Health Sciences Library has been the Index Medicus for the Eastern Mediterranean Region which was initiated in response to an urgent and acute need to index journals from the Region in the absence of the vast majority of these journals in international indexing and abstracting services such as MEDLINE. It aimed to map the health and biomedical literature in the Region and to put it on the global map, thus increasing the exposure of this literature at national, regional and global levels. One of the objectives of the IMEMR has been to assist member states to start their own national health and biomedical bibliographies. In 1987, the IMEMR started to index retrospectively articles published in health science journals in the Region since 1984. This process continued until 1997, by which time all articles in all the journals received had been indexed. By 1999, the backlog of journal articles had been completed, and the Index was published in five printed volumes. The final printed version of the Index was published in 1996 [5].

Since 1999, the Index has been published as a quarterly Current Contents bulletin. The 1987 Index covered 70 journals from the Region, while today the database covers 408 journals [6] from 19 countries. The IMEMR is one of the major elements of the World Health Organization's Global Health Library (GHL) project [7], which will provide first step access to reliable health information in paper form, electronic form, and other media.

The Regional Office encourages and supports electronic publishing and Open Access to health sciences journals in the Region. Out of the 408 journals indexed in the IMEMR, 181 journals (44.5%) are available on the Internet at the time of analysis as provided on the portal [8]. These journals are available on a free of charge basis. It should be noted, however, that only three journals are available in electronic format without a printed paper equivalent. Table 1 shows the distribution of number of journals and articles in countries.

**Table 1:** EMR health and biomedical journals

**Country**	**Total No. of Journals in the IMEMR**	**Total No. of articles in the IMEMR**	**%**	**Total No. of online journals**	**%**
	**N = 408**	**N = 91,249**		**N = 181**	
Bahrain	3	1,298	1.4	1	0.6
Egypt	115	39,578	43.5	22	12.0
Iraq	30	2,228	2.5	1	0.6
Islamic Republic of Iran	101	4,429	4.8	66	36.5
Jordan	5	1,139	1.3	4	2.1
Kuwait	4	2,072	2.3	3	1.7
Lebanon	9	2,140	2.3	5	2.7
Libyan Arab Jamahiriya	5	384	0.4	2	1.1
Morocco	6	1,404	1.5	0	0.0
Oman	2	675	0.7	1	0.6
Pakistan	63	16,373	18.0	53	29.3
Palestine	3	85	0.1	2	1.1
Qatar	4	715	0.8	2	1.1
Saudi Arabia	21	9,324	10.2	9	5.0
Sudan	9	369	0.4	2	1.1
Syrian Arab Republic	9	926	1.0	2	1.1
Tunisia	8	4,575	5.1	1	0.6
Untied Arab Emirates	3	992	1.0	3	1.6
Yemen	7	107	0.1	0	
Other non EMR countries	8	1577	1.7	1	0.6
Regional journals	1	859	0.9	1	0.6

The Index is managed by two full-time staff who work for the unit of Library and Information Networks as part of the Knowledge Management and Sharing Department. The actual work of indexing, data entry, proofreading and quality control is implemented through outsourcing to qualified health and biomedical professionals. An annual budget of US$ 75,000 is allocated by the Regional Office to fund the compilation, maintenance and publishing of the Index.

## Coverage and indexing policy of the IMEMR

### Geographic coverage

The geographic coverage of the IMEMR is the WHO Eastern Mediterranean Region, which comprises 22 countries as listed in Table 1. Prior to year 2000 seven journals published in European countries were also indexed due to the fact that they specialise in health and biomedicine in the Region. Indexing of journals published in Cyprus was stopped in 2004 after the country was reassigned to the WHO European Region.

### Subject coverage

The subject coverage of the IMEMR includes all public health topics, medicine and all its subspecialties, environmental health, dentistry, pharmaceutics, nursing, health management and administration and veterinary sciences. Excluded from the above are all magazines published for purely commercial promotion or trade purposes. News bulletins and newsletters that do not carry scientific and technical information are excluded from indexing in the IMEMR.

### Type of material

The Index exclusively covers peer-reviewed journals, which are defined as publications that contain scholarly articles written either by professors, researchers, or experts in a subject area and are subject to peer-review. An abstract and a bibliography often appear with each article [9].

### Language coverage

The major languages used in publishing of health and biomedical journals in the Region are covered. These are Arabic, English, Farsi and French. Other minor languages are also covered as long as Arabic, English or French abstracts are also available.

### Selection criteria of journals

The three major selection criteria are applied through initial screening by the library staff and after seeking the technical opinion of at least two scientists (programme directors and/or regional advisors) and the Regional Director's Senior Policy Advisor. These criteria are:

1. Subject specialization. The journal should cover health and biomedical sciences;

2. Geographic coverage. The journal should be published in one of the 22 countries of the Region;

3. Peer review. The journal should clearly state that it is a peer-reviewed journal, has an editorial board and an editorial policy.

### Indexing policy and procedures

The United States National Library of Medicine Medical Subject Headings (MeSH) [10] are used for indexing articles for the IMEMR. Indexers are provided with training on indexing techniques and policies. Each indexer is provided with a copy of the three volumes of Medical Subject Headings, in addition to access to the online bilingual (Arabic-English) version of the list [11]. To facilitate data entry and to eliminate spelling and typographical mistakes in the database, an electronic copy of the Medical Subject Headings was loaded onto the database which links the subject fields in the record to the Medical Subject Headings. Data entry clerks need only to pick from the list. The system excludes terms not listed in the Medical Subject Headings. In addition to main subject headings at least five indexing terms are assigned for each record. Categories are also added for male/female, human/animal, infant/adult, age group, case report/comparative study, etc. as per the indexing policies of the MEDLARS database. Abstracting services have been added to the database since 2005 for all newly added records. Retrospective abstracting was also initiated in the same year and a total of 39,109 abstracts (43% of the articles) have been added.

Medical doctors are employed for indexing articles in the IMEMR. The indexing process is composed of four steps, which are implemented by three different indexers:

1) Subject analysis and selection of indexing terms;

2) Revision of indexing terms assigned to each article;

3) Data entry of records in the database (by scientific secretaries);

4) Proofreading of data entered and quality control.

All entries in the database are in English. This requires translation of titles of articles from Arabic or French. The language of the original articles is recorded in the database. An author authority file has been developed to check standard transliteration of author's names.

## Publishing and distribution of the IMEMR

The target users of the Index include:

1. Medical librarians who act as intermediaries for researchers;

2. Health and biomedical researchers, scientists and academicians;

3. Health policy makers and managers to inform policy ;

4. Researchers and specialists of scientometrics to map scientific production.

Since 1999 the Index Medicus for the Eastern Mediterranean Region has been published in four formats:

1. Printed Current Contents. 1,300 copies of the quarterly Current Contents bulletin are produced by the Regional Office's printing facilities and distributed to libraries, journals, publishers, researchers, medical colleges, WHO Representatives' offices, Ministries of Health and other names/institutions on the Master Mailing List;

2. Electronic Current Contents. The quarterly Current Contents bulletin is also published on the Regional Office Intranet as a current awareness service for the Regional Office staff and field offices. The latest issue of the bulletin is also published on the Regional Office's website [12];

3. Web version. The full database is published on the Internet as part of the Regional Office's home page of health information support services [13];

4. CD-ROM version. The full database is also published on CD-ROM on a six-monthly basis and distributed to health sciences libraries in the Region.

## Technology platform and search engine

The United Nations Educational, Scientific and Cultural Organization (UNESCO) WINISIS (Integrated Set of Information Systems, Windows version) computerized information retrieval package is used to manage the Index database. The search engine developed by the WHO – Latin American and Caribbean Centre on Health Sciences Information is used to publish the database on the web and to provide full search capabilities to all the data elements in the database. The data elements comply with the UNESCO Common Communication Format and the Dublin Core. Search results may be displayed in different formats according to user needs. These can be copied to a file for further processing, and either full details or short records can be displayed.

The IMEMR is distributed free of charge, regardless of the format or the recipient. Country or subject-specific subsets of the database are published and distributed upon request or for specific occasions or health topic websites of the Regional Office, such as malaria, tuberculosis, etc.

The IMEMR has been made searchable through commercial search engines, including Google Scholar. This facility has allowed searching the database without having to go into its search interface, as all citations in the database are already indexed by Google.

## Bibliometric indicators in the IMEMR

### Countries contributing to the Index

Source journals in the Index are published in the Asian and African parts of the Region. At the time of writing, the Index includes 91,249 articles from 408 journals. The Region itself is divided in two large groups of countries (7 in Africa and 15 in Asia). The Index includes articles from eight journals fully dedicated to Middle East or Arabic medical literature. These are published in Italy (1), Germany (3), Turkey (1), Cyprus (1), and United Kingdom (2). These eight journals have contributed up to year 2004 a total of 1592 articles in the Index. A list of these journals published outside the Region appears as an annex to the main list of indexed journals [14]. The Eastern Mediterranean Health Journal, which is a regional journal has contributed 859 articles in the Index. Two countries in Africa (Djibouti and Somalia) and one country in Asia (Afghanistan) have not contributed any articles to the Index. Evidence shows that health and biomedical publishing is directly linked to the state of socioeconomic development and political situation of the country [15-21]. The three countries that have contributed no articles are among the least developed in the world. The data show that five countries in Africa contributed 52% of the articles in the database with two countries no contributions while 14 countries in Asia contributed 45% with one country with no contributions. Two large countries exist in the Region. Egypt (in Africa) with, a population of 72 million contributed 45% of the total database or 86% of the African contribution. Pakistan (156 million population) [22], the other large country in the Region contributed 17% of the total database or 39% of the Asian part of the database.

### Language distribution of articles

English is the dominant language of health and biomedical research in the Region. 91% of the articles indexed in the IMEMR are in English. English dominates for a number of reasons. It is the predominant language of scientific communication in many of the countries of the Region and in order to reach an international readership, authors tend to publish in English. 6% of the articles are in French originating mainly from North African countries. Other represented languages are Arabic (1.6%) and Farsi (1.3%), with a very small number of articles in Urdu (0.01%).

### Availability of English abstracts in IMEMR

It is unfortunate that at the inception of the IMEMR over 20 years ago, abstracting was not one of the services offered. Retrospective addition of abstracts started in 2005. All new citations in the database include abstracts as part of their bibliographic record. Priority has been given to the most recent journals and to journal articles that have original English abstracts.

### Date of publication of articles

The steady increase in the number of articles added to the database reflects the increasing number of journals covered in the database which has increased as indicated earlier from 70 in 1984 to 408 titles in 2008. The highest number of articles indexed is between 1992 and 1999 as by end of 1999 major effort was exerted to index journals retrospectively to avoid delay of publishing the Index.

### Top authors in EMR journals as reflected in the Index

Since Egypt contributes a major proportion of the database, Egyptian authors naturally came on top of the list, ranking according to the total number of articles of which one is an author or a co-author. Ten authors from Pakistan were among the top 20 on the list. This ranking maybe used to identify consultants, researchers or educators for a given subject. It will continue to help in forming networks of researchers in various areas in the Region.

### Top subjects in EMR medical journals as reflected in the Index

Subjects covered by the Index include all health and biomedical topics, environmental health, pharmacy, dentistry and veterinary sciences. The ten most popular topics (MeSH headings) covered were blood, surgery, hospital, pathology, **epidemiology (8%)**, liver, public health, child, women and pregnancy, which together make up 90% of the records in the database.

### Type of articles indexed in IMEMR

The vast majority of published materials are journal articles, clinical trials, and/or case reports, which together represent 91% of the total articles in the database.

## IMEMR and other international indexing and abstracting services

As indicated earlier, the unique feature of the IMEMR is its combination of geographic (Eastern Mediterranean Region), subject (health and biomedical) and publication type (journal articles) coverage. Overlap with other international databases is minimal as no other database has the same mandate as the IMEMR. MEDLINE, as one of the most internationally recognized databases covering health and biomedical subjects, has articles from 62 out of 408 (or 15.4%) journals from the Region. The selection criteria of MEDLINE and its indexing policies do not seem to allow for the majority of EMR journals to be indexed in the database. MEDLINE's criteria cover subject suitability, quality of contents, quality of editorial work, production quality, selection methods of proposed articles, type of contributions, language and geographic coverage. EMBASE is probably the second largest database covering health and biomedical literature. EMBASE includes articles from 28 out of 408 (7%) journals from the Region. The list of journals in the IMEMR database indicates the journals that are indexed in MEDLINE and/or EMBASE. Among the other databases that cover major health (reproductive health in particular) topics from the Region is POPLINE, which describes itself as the world's largest database on reproductive health. It provides more than 350,000 citations, with abstracts of scientific articles, reports, books, and unpublished reports in the field of population, family planning, and related health issues. The POPLINE database includes selected articles from EMR journals that meet its criteria. It does not, however, index complete journals from the Region.

## WHO support to health and biomedical journals in the Region

The Regional Office has been providing both direct and indirect support to medical journal publishing in the Region. Among the different forms of support provided are:

1. Coverage of these journals in the IMEMR, which has exposed them to the international medical research and academic community. The IMEMR has been a tool to map the literature and guide researchers from all over the world to journal articles in the Region;

2. Agreement with selected journals to reprint their articles in the East Mediterranean Health Journal (EMHJ) [23] and vice versa. This has provided a great opportunity for the best articles to be indexed in the MEDLINE, since the EMHJ itself is indexed there. The Editorial Board of the EMHJ conducts the selection and recommends articles for reprinting;

3. A series of training courses has been organized in a number of countries to train national staff of medical journals in peer reviewing, editing, literature searching, data collection, data analysis, case reporting, proposal writing, citation process, presentation, etc.

4. Direct financial support to health and biomedical journals, either through subsidising subscription to these journals and their distribution through the Regional Office's channels or direct financing to assist the journal in setting up desktop publishing facilities (computers, printers, scanners, photocopiers), and/or supporting the cost of printing of journals for a limited period of time;

5. Provision of financial support to medical indexing and abstracting projects in EMR countries, to help them develop national medical bibliographies;

6. Provision of document delivery services based on articles from the Index free of charge, which has helped access to medical journals from the Region.

7. The Index has constituted a platform for networking and collaboration among editors and publishers of biomedical journals in the Region. This has resulted in the creation of the Eastern Mediterranean Association of Medical Editors (EMAME). EMAME convened three regional conferences and organized a series of training workshops for journals' editors.

## Constraints and lessons learned

Sustaining (compiling, maintenance and publishing) the Index has not been without difficulties and constraints. These have included:

• Shortage of funding as this project has to compete with other more urgent activities such as journals subscriptions, training, collection development, and building information infrastructure;

• Lack of trained indexers who are both subject experts and have the indexing skills;

• Many journals at the early stage of indexing did not believe in the value of indexing which resulted in irregular provision of journals for indexing;

• Many journals are published on an irregular basis or cease after publishing few issues;

• The technological platform has to be upgraded and migrated few times over the last two decades. Quality control of data has been the most challenging task.

Many attempts have been made to move the indexing of articles to countries to be totally managed by national institutions. This has not been possible due to lack of resources and expertise in many countries. This remains a long term objective that requires building capacity at national level to institutionalize the service.

## Future directions for the IMEMR

The Index has been maintained professionally for over 20 years and has received strong support from the Regional Office's leadership. It has become a cornerstone in the knowledge management and sharing programme. It has served many useful purposes, and has received recognition by information users, journals' publishers and the information and library community.

Future plans for developing the IMEMR include the following components:

1. **Adding abstracts to the database**. As indicated, about half of the articles listed on the database have an original abstract, which means that only data entry and proofreading costs need to be met. The other half would require preparation of abstracts by biomedical professionals. Adding the abstracts would elevate the IMEMR to a higher level of professionalism, quality and usefulness.

2. **Digitization and imaging of articles**. A number of journals, especially in Egypt, have already started creating images of articles and publishing them as PDF (Portable Document Format) files on the Internet. Other journals have started publishing their articles on the Internet in HTML (Hyper Text Mark up Language) format as is clear from the statistics (181 journals out of 408 are electronically available) shown above. A third group of journals is already included in other projects such as EXTRAMED which was created in 1993 on the initiative of the World Health Organization (WHO), which brought together the publishers of over 290 biomedical journals from all over the world into the ExtraMED Consortium. [24]. An assessment is needed of how much it will cost and the timeframe needed to develop a plan and a final decision.

3. **Adding monographs and other formal publications**. This may include books, theses, handbooks, etc. A project to create a regional bibliography of Arabic medical books has already been launched [25].

4. **Adding current medical research **as a separate service form the Index and as part of WHO International Clinical Trials Registry Platform aiming to ensure that a complete view of research is accessible to all those involved in health care decision making.

5. **Adding a search facility to allow bilingual searching of the database **in both Arabic and English using the National Library of Medicine Medical Subject Headings of the National Library of Medicine.

6. **Linking articles to parent institutions of authors **for citation analysis. The majority of records in the database include only the titles and address of the journals but not the author's institution. Adding this feature would allow cross-linking and networking between institutions and researchers.

## Annex 1 List of journals indexed in IMEMR

A list of journals indexed in IMEMR, with their ISSN and country of publication is provided as Additional file 1. Those journals that are also indexed in MEDLINE and EMBASE are also indicated.

## Abstracts in non-English languages

The abstract of this paper has been translated into the following languages by the following translators (names in brackets):

• Arabic (Dr. Kassem Sara) [see Additional file 2]

• Chinese – simplified characters (Mr. Isaac Chun-Hai Fung) [see Additional file 3]

• Chinese – traditional characters (Mr. Isaac Chun-Hai Fung) [see Additional file 4]

• French (Ms. Annick Bórquez) [see Additional file 5]

• Spanish (Ms. Annick Bórquez) [see Additional file 6]

## Competing interests

The author declares that he has no competing interests.

## Supplementary Material

Additional file 1 Annex 1: List of journals indexed in IMEMR Click here for file

Additional file 2 Abstract in Arabic Click here for file

Additional file 3 Abstract in Chinese – Simplified characters Click here for file

Additional file 4 Abstract in Chinese – Traditional characters Click here for file

Additional file 5 Abstract in French Click here for file

Additional file 6 Abstract in Spanish Click here for file
