# Supplementary material for: Index medicus for the Eastern Mediterranean region
Source: Emerg Themes Epidemiol. 2008 Sep 30;5:14. doi: 10.1186/1742-7622-5-14 (PMC2565659; doi:10.1186/1742-7622-5-14)
Supplement: Additional file 1 — Annex 1: List of journals indexed in IMEMR [file 1742-7622-5-14-S1.pdf]

ANNEX 1
LIST OF JOURNALS INDEXED IN IMEMR
JOURNALS INDEXED IN MEDLINE AND/OR EMABSE ARE MARKED (√)

No	INDEXED IN MEDLINE	INDEXED IN EMBASE	ISSN	Journal Title	Country
1	X	X	1687-1693	AAMJ - Al-Azhar Assiut Medical Journal	EGYPT
2	X	X	1683-8068	ACES - Actualites Cliniques et Scientifiques	LEBANON
3	√	X	0044-6025	Acta Medica Iranica	ISLAMIC REPUBLIC OF IRAN
4	X	X	1561-4174	Advances in Cognitives Sciences	ISLAMIC REPUBLIC OF IRAN
5	X	X	1687-224X	Afro - Arab Liver Journal	EGYPT
6	X	X	1687-1030	Ain-Shams Journal of Forensic Medicine and Clinical Toxicology	EGYPT
7	√	X	0002-2144	Ain-Shams Medical Journal	EGYPT
8	X	X	N/A	AJAIC - Alexandria Journal of Anaesthesia and Insentive Care	EGYPT
9	√	X	1110-0400	Al Azhar Medical Journal	EGYPT
10	X	X	N/A	Al-Azhar Journal of Dental Science	EGYPT
11	√	X	1110-015X	Alexandria Dental Journal	EGYPT
12	X	X	N/A	Alexandria Journal of Food Science and Technology	EGYPT
13	X	X	N/A	Alexandria Journal of Pediatrics	EGYPT
14	√	X	1110-1792	Alexandria Journal of Pharmaceutical Sciences	EGYPT
15	X	X	1110-2047	Alexandria Journal of Veterinary Science	EGYPT
16	√	X	0516-5849	Alexandria Medical Journal [The]	EGYPT
17	X	X	1810-9543	Al-Kindy College Medical Journal	IRAQ
18	X	X	N/A	Al-Majallah Al-Tibbiya Al-Arabiayh	SYRIAN ARAB REPUBLIC
19	X	X	1815-0993	Almustansiriya Journal of Pharmaceutical Sciences	IRAQ
20	X	X	N/A	Al-Quds Medical Journal	PALESTIN
21	X	X	1563-3241	Annals Abbassi Shaheed Hospital and Karachi Medical and Dental College	PAKISTAN
22	X	X	1815-2643 Online	Annals of Alquds Medicine	PALESTIN
23	X	X	N/A	Annals of Jinnah Postgraduate Medical Centre - Karachi	PAKISTAN
24	X	X	1684-6680	Annals of King Edward Medical College	PAKISTAN

No	INDEXED IN MEDLINE	INDEXED IN EMBASE	ISSN	Journal Title	Country
25	X	X	1687-4137	Annals of Pediatric Surgery	EGYPT
26	√	√	0256-4947	Annals of Saudi Medicine	SAUDI ARABIA
27	X	X	0027-1446	Annals of the College of Medicine - Mosul	IRAQ
28	X	√	1817-1737	Annals of Thoracic Medicine	SAUDI ARABIA
29	X	X	N/A	Arab Dental Journal	LEBANON
30	X	X	1608-8352	Arab Journal for Food and Nutrition	BAHRAIN
31	√	X	1110-6875	Arab Journal of Biotechnology	EGYPT
32	X	X	1110-1822	Arab Journal of Laboratory Medicine [The]	EGYPT
33	X	X	1683-0369	Arab Journal of Pharmaceutical Sciences	SYRIAN ARAB REPUBLIC
34	X	X	1016-8923	Arab Journal of Psychiatry [The]	JORDAN
35	X	X	N/A	Arabic Journal of Forensic Medicine and Criminal Science	SYRIAN ARAB REPUBLIC
36	√	X	0020-2509	Archives de l'Institut Pasteur de Tunis	TUNISIA
37	√	√	1029-2977	Archives of Iranian Medicine	ISLAMIC REPUBLIC OF IRAN
38	X	X	1728-6506	Armaghane-danesh	ISLAMIC REPUBLIC OF IRAN
39	X	X	1735-3955	ARYA Therosclerosis	ISLAMIC REPUBLIC OF IRAN
40	X	X	1687-2193	ASJOG - Ain Shams Journal of Obstetrics and Gynecology	EGYPT
41	X	X	N/A	ASNJ - Alexandria Scientific Nursing Journal	EGYPT
42	X	X	1110-0494	Assiut Medical Journal	EGYPT
43	X	X	1735-1936	Audiology	ISLAMIC REPUBLIC OF IRAN
44	X	√	1012-8298	Bahrain Medical Bulletin	BAHRAIN
45	X	X	N/A	Basrah Journal of Surgery	IRAQ
46	X	X	1110-208X	Benha Medical Journal	EGYPT
47	X	X	1026-6399	Bina Journal of Ophthalmology	ISLAMIC REPUBLIC OF IRAN
48	X	X	N/A	Biomedica	PAKISTAN
49	X	X	1027-9520	Blood	ISLAMIC REPUBLIC OF IRAN
50	X	X	0851-8238	Bulletin epidemiologique	MOROCCO
51	√	X	1110-0834	Bulletin of Alexandria Faculty of Medicine	EGYPT

No	INDEXED IN MEDLINE	INDEXED IN EMBASE	ISSN	Journal Title	Country
52	X	X	N/A	Bulletin of Alexandria Thoracic Association	EGYPT
53	√	X	0007-4845	Bulletin of Endemic Diseases - Baghdad	IRAQ
54	X	X	1110-0931	Bulletin of Faculty of Pharmacy - Cairo University	EGYPT
55	X	X	1110-6611	Bulletin of Faculty of Physical Therapy - Cairo University	EGYPT
56	√	X	1110-0036	Bulletin of High Institute of Public Health	EGYPT
57	X	X	1110-0052	Bulletin of Pharmaceutical Sciences - Assiut University	EGYPT
58	X	X	0568-9619	Bulletin of the Faculty of Science - University of Alexandria	EGYPT
59	X	X	N/A	Bulletin of the Kuwait Institute for Medical Specialization	KUWAIT
60	X	X	N/A	Bulletin of the National Nutrition Institute of the Arab Republic of Egypt	EGYPT
61	X	X	1110-0591	Bulletin of the National Research Centre	EGYPT
62	√	X	1110-0982	Bulletin of the Ophthalmological Society of Egypt	EGYPT
63	X	X	0300-5291	Cahier Medicaux de Tunisie	TUNISIA
64	X	X	0528-7944	Challenge - Quarterly [The]	PAKISTAN
65	X	X	1110-8681	Childhood and Development	EGYPT
66	X	√	0891-8929	Clinical Diabetes	EGYPT
67	X	X	0301-7265	Community Medicine	PAKISTAN
68	√	X	N/A	Damascus University Journal for Health Sciences	SYRIAN ARAB REPUBLIC
69	X	√	1560-8115	DARU - Journal of Faculty of Pharmacy Tehran University of Medical Sciences	ISLAMIC REPUBLIC OF IRAN
70	X	X	1369-7501	Diabetes Digest	PAKISTAN
71	√	X	1026-3772	Dirasat	JORDAN
72	X	X	1735-3327	DRJ - Dental Research Journal	ISLAMIC REPUBLIC OF IRAN
73	X	X	1040-2312	Eastern Mediterranean Region Drugs Digest	EGYPT
74	X	X	1014-2347	Eastern Mediterranean Region Epidemiological Bulletin	EGYPT
75	√	X	0070-9484	EDJ - Egyptian Dental Journal	EGYPT
76	X	X	1110-2144	Egyptian Journal of Anatomy [The]	EGYPT
77	X	X	1012-5558	Egyptian Journal of Biomedical Engineering	EGYPT
78	X	X	1110-8525	Egyptian Journal of Biophysics and Biomedical Engineering	EGYPT
79	X	X	0449-2285	Egyptian Journal of Chemistry	EGYPT
80	X	X	1110-1865	Egyptian Journal of Community Medicine	EGYPT

No	INDEXED IN MEDLINE	INDEXED IN EMBASE	ISSN	Journal Title	Country
81	X	X	1110-7650	Egyptian Journal of Dermatology and Andrology	EGYPT
82	X	X	N/A	Egyptian Journal of Diabetes [The]	EGYPT
83	X	X	1110-0613	Egyptian Journal of Food Science	EGYPT
84	√	X	0046-161X	Egyptian Journal of Genetics and Cytology	EGYPT
85	√	X	1110-0559	Egyptian Journal of Histology [The]	EGYPT
86	X	X	N/A	Egyptian Journal of Hospital Medicine [The]	EGYPT
87	X	X	1110-8630	Egyptian Journal of Medical Human Genetics [The]	EGYPT
88	X	X	1110-5593	Egyptian Journal of Medical Laboratory Sciences	EGYPT
89	X	X	1110-2179	Egyptian Journal of Medical Microbiology	EGYPT
90	X	X	0022-2704	Egyptian Journal of Microbiology	EGYPT
91	X	X	N/A	Egyptian Journal of Neonatology [The]	EGYPT
92	X	X	N/A	Egyptian Journal of Nutrition	EGYPT
93	√	X	0258-7556	Egyptian Journal of Occupational Medicine	EGYPT
94	X	X	0301-5068	Egyptian Journal of Pharmaceutical Sciences [The]	EGYPT
95	X	X	0301-8660	Egyptian Journal of Physiological Sciences	EGYPT
96	X	X	1110-1105	Egyptian Journal of Psychiatry [The]	EGYPT
97	X	X	1110-7278	Egyptian Journal of Schistosomiasis and Infectious and Endemic Diseases	EGYPT
98	X	X	1110-1121	Egyptian Journal of Surgery [The]	EGYPT
99	X	X	1110-5712	Egyptian Journal of Urology	EGYPT
100	X	X	1110-0222	Egyptian Journal of Veterinary Science	EGYPT
101	X	X	1687-1278	Egyptian Medical Journal of the National Research Center	EGYPT
102	X	X	N/A	Egyptian Orthodontic Journal	EGYPT
103	X	X	1110-1148	Egyptian Orthopaedic Journal [The]	EGYPT
104	X	X	0013-2438	Egyptian Pharmaceutical Journal [National Research Center]	EGYPT
105	√	X	N/A	Egyptian Population and Family Planning Review [The]	EGYPT
106	X	X	1110-161X	Egyptian Rheumatology and Rehabilitation	EGYPT
107	X	X	1687-3815	Egyptian Science Magazine [The]	EGYPT

No	INDEXED IN MEDLINE	INDEXED IN EMBASE	ISSN	Journal Title	Country
108	√	X	1687-1502	EJB - Egyptian Journal of Biochemistry and Molecular Biology	EGYPT
109	X	X	N/A	EJENTAS - Egyptian Journal of ENT and Allied Sciences	EGYPT
110	X	X	1110-2446	El-Minia Medical Bulletin	EGYPT
111	√	√	1020-3397	EMHJ - Eastern Mediterranean Health Journal	EGYPT
112	X	√	0250-6882	Emirates Medical Journal	UNITED ARAB EMIRATES
113	√	X	1110-0095	EMJ - Egyptian Medical Journal [The]	EGYPT
114	X	X	0254-7198	Garyounis Medical Journal	LYBIAN ARAB JAMAHIRIYA
115	√	X	1110-6638	Gazette of the Egyptian Paediatric Association [The]	EGYPT
116	X	X	N/A	Gezira Journal of Health Sciences	SUDAN
117	X	X	1819-7973	GJMS – Gomal Journal of Medical Sciences	PAKISTAN
118	X	X	1560-7186	Govaresh	ISLAMIC REPUBLIC OF IRAN
119	X	X	N/A	Gulf Journal of Dermatology and Venereology [The]	QATAR
120	X	X	1561-252X	HAKIM Research Journal	ISLAMIC REPUBLIC OF IRAN
121	√	X	0250-7188	Hamdard Medicus	PAKISTAN
122	X	X	1735-2215	HAYAT - The Journal of Faculty of Nursing and Midwifery	ISLAMIC REPUBLIC OF IRAN
123	X	X	1735-1847	Health Information Management	ISLAMIC REPUBLIC OF IRAN
124	X	X	1014-9899	Health Services Journal of the Eastern Mediterrenean Region	EGYPT
125	X	X	N/A	Heart Views	QATAR
126	X	X	1735-143X	Hepatitis Monthly	ISLAMIC REPUBLIC OF IRAN
127	√	√	1028-852X	IBJ - Iranian Biomedical Journal	ISLAMIC REPUBLIC OF IRAN
128	X	X	N/A	IDEES - Revue de Perfectionnement Medical et Paramedical	TUNISIA
129	X	X	1735-4668	IJCN - Iranian Journal of Child Neurology	ISLAMIC REPUBLIC OF IRAN
130	X	X	1683-4844	IJEM - Iranian Journal of Endocrinology and Metabolism	ISLAMIC REPUBLIC OF IRAN
131	X	X	1735-1243	IJHOBMT - International Journal of Hematology-Oncology and Bone Marrow Transplantation	ISLAMIC REPUBLIC OF IRAN
132	√	X	1735-1383	IJI - Iranian Journal of Immunology	ISLAMIC REPUBLIC OF IRAN
133	√	X	1735-8582	IJKD – Iranian Journal of Kidney Diseases	ISLAMIC REPUBLIC OF IRAN
134	X	X	1608-9359	IJME - Iranian Journal of Medical Education	ISLAMIC REPUBLIC OF IRAN
135	X	√	0253-0716	IJMS - Iranian Journal of Medical Sciences	ISLAMIC REPUBLIC OF IRAN

No	INDEXED IN MEDLINE	INDEXED IN EMBASE	ISSN	Journal Title	Country
136	X	X	1735-5087	IJO - Iranian Journal of Orthodontics	ISLAMIC REPUBLIC OF IRAN
137	X	X	1735-0328	IJPR - Iranian Journal of Pharmaceutical Research	ISLAMIC REPUBLIC OF IRAN
138	X	X	0304-4564	IMJ - Iraqi Medical Journal	IRAQ
139	X	X	1027-0299	Infectious Disease Journal of Pakistan	PAKISTAN
140	X	√	1606-7754	International Journal of Diabetes and Metabolism	UNITED ARAB EMIRATES
141	X	X	1726-9148	International Journal of Endocrinology and Metabolism	ISLAMIC REPUBLIC OF IRAN
142	X	X	1735-6865	International Journal of Environmental Research	ISLAMIC REPUBLIC OF IRAN
143	X	X	1735-1472	International Journal of Environmental Science and Technolgy	ISLAMIC REPUBLIC OF IRAN
144	X	X	1810-0155	International Journal of Pathology	PAKISTAN
145	X	X	1816-0581	IOJ - Iraqi Orthodontic Journal	IRAQ
146	X	X	1608-8360	IPMJ - Iraqi Postgraduate Medical Journal	IRAQ
147	X	X	1735-8612	Iran Journal of Medical Microbiology	ISLAMIC REPUBLIC OF IRAN
148	√	X	1025-0581	Iran Journal of Nursing	ISLAMIC REPUBLIC OF IRAN
149	√	√	1735-1502	Iranian Journal of Allergy, Asthma and Immunology	ISLAMIC REPUBLIC OF IRAN
150	X	X	1735-7179	Iranian Journal of Arthropod-Borne Diseases	ISLAMIC REPUBLIC OF IRAN
151	√	X	1608-6015	Iranian Journal of Basic Medical Sciences	ISLAMIC REPUBLIC OF IRAN
152	√	X	1728-3043	Iranian Journal of Biotechnology	ISLAMIC REPUBLIC OF IRAN
153	X	X	N/A	Iranian Journal of Clinical Infectious Diseases	ISLAMIC REPUBLIC OF IRAN
154	X	X	0021-082X	Iranian Journal of Dermatology	ISLAMIC REPUBLIC OF IRAN
155	X	√	1726-7544	Iranian Journal of Diabetes and Lipid Disorders	ISLAMIC REPUBLIC OF IRAN
156	X	X	1735-1979	Iranian Journal of Environmental Health Science and Engineering	ISLAMIC REPUBLIC OF IRAN
157	X	X	N/A	Iranian Journal of Epidemiology	ISLAMIC REPUBLIC OF IRAN
158	√	X	1735-8094	Iranian Journal of Fertility and Sterility	ISLAMIC REPUBLIC OF IRAN
159	X	√	1681-2824	Iranian Journal of Nuclear Medicine	ISLAMIC REPUBLIC OF IRAN
160	X	X	N/A	Iranian Journal of Obstetric, Gynecology and Infertility [The]	ISLAMIC REPUBLIC OF IRAN
161	X	X	N/A	Iranian Journal of Ophthalmic Research	ISLAMIC REPUBLIC OF IRAN
162	X	X	1560-1293	Iranian Journal of Otorhinolaryngology	ISLAMIC REPUBLIC OF IRAN

No	INDEXED IN MEDLINE	INDEXED IN EMBASE	ISSN	Journal Title	Country
163	X	X	1735-7020	Iranian Journal of Parasitology	ISLAMIC REPUBLIC OF IRAN
164	X	X	1018-4406	Iranian Journal of Pediatrics	ISLAMIC REPUBLIC OF IRAN
165	X	X	1735-4587	Iranian Journal of Psychiatry	ISLAMIC REPUBLIC OF IRAN
166	X	X	1024-0047	Iranian Journal of Psychiatry and Clinical Psychology (ANDEESHEH VA RAFTAR)	ISLAMIC REPUBLIC OF IRAN
167	√	X	0304-4556	Iranian Journal of Public Health	ISLAMIC REPUBLIC OF IRAN
168	X	√	1728-4554	Iranian Journal of Radiation Research	ISLAMIC REPUBLIC OF IRAN
169	X	X	1735-1065	Iranian Journal of Radiology	ISLAMIC REPUBLIC OF IRAN
170	X	X	N/A	Iranian Journal of Reproductive Medicine	ISLAMIC REPUBLIC OF IRAN
171	X	X	1728-1997	Iranian Journal of Veterinary Research	ISLAMIC REPUBLIC OF IRAN
172	X	X	N/A	Iranian Rehabilitation Journal	ISLAMIC REPUBLIC OF IRAN
173	X	X	N/A	Iraqi Army Medical Journal	IRAQ
174	X	X	N/A	Iraqi Journal of Agriculture	IRAQ
175	X	X	N/A	Iraqi Journal of Biotechnology	IRAQ
176	X	X	1684-5382	Iraqi Journal of Community Medicine	IRAQ
177	X	X	1681-6579	Iraqi Journal of Medical Sciences	IRAQ
178	X	X	N/A	Iraqi Journal of Microbiology	IRAQ
179	X	X	N/A	Iraqi Journal of Pharmaceutical Sciences	IRAQ
180	X	X	1814-0823	Iraqi Journal of Tropical Disease Researches	IRAQ
181	√	X	1561-4395	IRCMJ – Iranian Red Crescent Medical Journal	UNITED ARAB EMIRATES
182	√	X	1025-9589	JAMC - Journal of Ayub Medical College - Abbotabad - Pakistan	PAKISTAN
183	√	√	1015-6321	JBMS - Journal of the Bahrain Medical Society	BAHRAIN
184	√	√	1022-386X	JCPSP - Journal of the College of Physicians and Surgeons Pakistan	PAKISTAN
185	X	X	1735-2150	JDT - Journal of Dentistry Tehran University of Medical Sciences	ISLAMIC REPUBLIC OF IRAN
186	X	X	N/A	JDUHS - Journal of the Dow University of Health Sciences	PAKISTAN
187	X	X	1303-667X	JISHIM - Journal of the International Society for the History of Islamic Medicine	SYRIAN ARAB REPUBLIC
188	X	X	1810-9632	JLDA - Journal of the Lebanese Dental Association	LEBANON
189	X	√	1729-0341	JLUMHS - Journal of the Liaquat University of Medical Health Sciences	PAKISTAN
190	X	X	N/A	JMJ - Jamahiriya Medical Journal	LYBIAN ARAB JAMAHIRIYA

No	INDEXED IN MEDLINE	INDEXED IN EMBASE	ISSN	Journal Title	Country
191	X	X	N/A	JMJ - Juba Medical Journal	SUDAN
192	X	X	1728-1962	JMR - Journal of Medical Research	ISLAMIC REPUBLIC OF IRAN
193	X	√	N/A 1011-4564	JMS - Journal of Medical Sciences	PAKISTAN
194	X	X	N/A	Joint Centre for Research in Prosthetics and Orthotics [The]	SAUDI ARABIA
195	X	X	N/A	JOPDAK - Journal of the Pakistan Dental Association Karachi	PAKISTAN
196	X	√	0446-9283	Jordan Medical Journal	JORDAN
197	X	X	1113-5667	Journal du Practicien	MOROCCO
198	X	X	N/A	Journal of Arak University of Medical Sciences - Rahavard Danesh	ISLAMIC REPUBLIC OF IRAN
199	X	X	1727-1789	Journal of Army University of Medical Sciences – J.A.U.M.S	ISLAMIC REPUBLIC OF IRAN
200	X	X	1561-4107	Journal of Babol University of Medical Sciences	ISLAMIC REPUBLIC OF IRAN
201	X	X	N/A	Journal of Basic and Applied Sciences	PAKISTAN
202	√	X	1608-6015	Journal of Basic Medical Sciences	IRAQ
203	X	X	N/A	Journal of Childhood Studies	EGYPT
204	X	X	N/A	Journal of Clinical Laboratory [The]	SYRIAN ARAB REPUBLIC
205	X	X	N/A	Journal of Community Medicine	IRAQ
206	X	X	1024-641X	Journal of Dentistry - Tehran University of Medical Sciences	ISLAMIC REPUBLIC OF IRAN
207	X	X	1728-3426	Journal of Dentistry, Shiraz University of Medical Sciences	ISLAMIC REPUBLIC OF IRAN
208	X	X	1812-7568	Journal of Dohuk University	IRAQ
209	√	X	0085-2406	Journal of Drug Research	EGYPT
210	X	X	1319-1683	Journal of Family and Community Medicine	SAUDI ARABIA
211	X	X	1562-4765	Journal of Gorgan University of Medical Sciences	ISLAMIC REPUBLIC OF IRAN
212	X	X	1110-0796	Journal of Hepatology, Gastroenterology and Infectious Diseases	EGYPT
213	X	X	1735-2363	Journal of Hygiene and Health	ISLAMIC REPUBLIC OF IRAN
214	X	X	1735-255X	Journal of Isfahan Dental School	ISLAMIC REPUBLIC OF IRAN
215	X	X	1027-7595	Journal of Isfahan Medical School	ISLAMIC REPUBLIC OF IRAN
216	X	X	1024-6428	Journal of Islamic Dental Association of Iran [The]	ISLAMIC REPUBLIC OF IRAN
217	X	X	1813-0410	Journal of Karbala University	IRAQ
218	X	X	1023-9510	Journal of Kerman University of Medical Sciences	ISLAMIC REPUBLIC OF IRAN

No	INDEXED IN MEDLINE	INDEXED IN EMBASE	ISSN	Journal Title	Country
219	X	X	1319-1004	Journal of King Abdulaziz University - Medical Sciences	SAUDI ARABIA
220	X	X	1110-6468	Journal of Legal Medicine and Forensic Sciences[The]	EGYPT
221	X	X	1560-9286	Journal of Mashad Dental School	ISLAMIC REPUBLIC OF IRAN
222	X	X	1561-4123	Journal of Mazandaran University of Medical Sciences	ISLAMIC REPUBLIC OF IRAN
223	X	X	1562-1073	Journal of Medical Council of Islamic Republic of Iran	ISLAMIC REPUBLIC OF IRAN
224	X	X	N/A	Journal of Medical Education	ISLAMIC REPUBLIC OF IRAN
225	X	√	1682-4474	Journal of Medical Sciences	PAKISTAN
226	X	√	1684-0240	Journal of Medicinal Plants	ISLAMIC REPUBLIC OF IRAN
227	X	√	N/A 1606-9331	Journal of Nephrology Urology and Transplantation	PAKISTAN
228	X	X	1561-3666	Journal of Qazvin University of Medical Sciences and Health Services [The]	ISLAMIC REPUBLIC OF IRAN
229	X	X	1562-272X	Journal of Shaheed Sadoughi University of Medical Sciences and Health Services	ISLAMIC REPUBLIC OF IRAN
230	X	X	1735-1448	Journal of Shahrekord University of Medical Sciences	ISLAMIC REPUBLIC OF IRAN
231	X	X	1681-4517	Journal of Surgery [The]	PAKISTAN
232	X	X	1735-5370	Journal of Tehran Heart Center [The]	ISLAMIC REPUBLIC OF IRAN
233	X	X	1561-0217	Journal of the Arab Board of Medical Specializations	SYRIAN ARAB REPUBLIC
234	X	X	N/A	Journal of the Arab Dentist	SYRIAN ARAB REPUBLIC
235	X	X	1812-1756	Journal of the Arab Neonatology Forum	LEBANON
236	√	X	1687-4293	Journal of the Arab Society for Medical Research	EGYPT
237	X	X	N/A	Journal of the College of Dentistry - Baghdad	IRAQ
238	X	X	N/A	Journal of the Dental School - Shahid Beheshti Medical Sciences University -	ISLAMIC REPUBLIC OF IRAN
239	√	X	0013-2411	Journal of the Egyptian Medical Association [The]	EGYPT
240	√	X	1110-0362	Journal of the Egyptian National Cancer Institute	EGYPT
241	√	X	N/A	Journal of the Egyptian Public Health Association [The]	EGYPT
242	X	X	N/A	Journal of the Egyptian Society of Endocrinology, Metabolism and Diabetes [The]	EGYPT
243	√	X	0258-3216	Journal of the Egyptian Society of Obstetrics and Gynecology [The]	EGYPT
244	√	X	1110-0583	Journal of the Egyptian Society of Parasitology	EGYPT
245	X	X	N/A	Journal of the Egyptian Society of Pharmacology and Experimental Therapeutics [The]	EGYPT
246	X	X	1110-127X	Journal of the Egyptian Society of Toxicology	EGYPT
247	√	X	0041-9419	Journal of the Faculty of Medicine - Baghdad	IRAQ

No	INDEXED IN MEDLINE	INDEXED IN EMBASE	ISSN	Journal Title	Country
248	X	X	N/A	Journal of the Faculty of Medicine - Shaheed Beheshti University of Medical Sciences and Health Services	ISLAMIC REPUBLIC OF IRAN
249	X	X	1022-646X	Journal of the Faculty of Veterinary Medicine - University of Tehran	ISLAMIC REPUBLIC OF IRAN
250	X	X	1110-0133	Journal of the Medical research Institute - Alexandria University	EGYPT
251	X	X	N/A	Journal of the Royal Medical Services	JORDAN
252	X	X	1319-9218	Journal of the Saudi Heart Association	SAUDI ARABIA
253	X	X	N/A	Journal of Tropical Nephro-Urology	YEMEN
254	X	X	1606-9366	Journal of Zanjan University of Medical Sciences and Health Services	ISLAMIC REPUBLIC OF IRAN
255	X	√	1560-9014	JPAD - Journal of Pakistan Association of Dermatologists	PAKISTAN
256	X	X	N/A	JPC - Journal of Pediatric Club	EGYPT
257	X	X	1680-2292	JPDA - Journal of the Pakistan Dental Association	PAKISTAN
258	X	X	1683-6928	JPIMS - Journal of Pakistan Institute of Medical Sciences	PAKISTAN
259	√	√	0030-9982	JPMA - Journal of Pakistan Medical Association	PAKISTAN
260	X	√	1013-5472	JPMI - Journal of Postgraduate Medicale Institute	PAKISTAN
261	√	X	1726-8710	JPPS - Journal of Pakistan Psychiatric Society	PAKISTAN
262	X	√	1735-1995	JRMS - Journal of Research in Medical Sciences	ISLAMIC REPUBLIC OF IRAN
263	X	X	1817-0242	JSP - Journal of Surgery Pakistan International	PAKISTAN
264	X	X	N/A	Kasr El Aini Journal of Surgery	EGYPT
265	X	X	N/A	Khartoum Pharmacy Journal	SUDAN
266	X	X	1369-3050	Kidney Forum	EGYPT
267	√	X	0023-5776	KMJ - Kuwait Medical Journal	KUWAIT
268	X	√	1608-7046	KOOMESH - Journal of the Samman University of Medical Sciences	ISLAMIC REPUBLIC OF IRAN
269	X	X	N/A	Kufa Medical Journal	IRAQ
270	X	X	N/A	Lahore Journal of Public Health	PAKISTAN
271	X	X	1561-3410	Lebanese Science Journal	LEBANON
272	X	X	N/A	Libyan Journal of Infectious Diseases [The]	LIBYAN ARAB JAMAHIRYA
273	X	X	1819-6357	LJM - Libyan Journal of Medicine	LIBYAN ARAB JAMAHIRYA
274	√	X	0023-9852	LMJ - Lebanese Medical Journal	LEBANON
275	√	X	0330-258X	Maghreb Medical	TUNISIA

No	INDEXED IN MEDLINE	INDEXED IN EMBASE	ISSN	Journal Title	Country
276	X	X	1110-1318	Mansoura Journal of Pharmaceutical Sciences	EGYPT
277	X	X	1110-211X	Mansoura Medical Journal	EGYPT
278	√	X	0025-4053	Maroc Medical	MOROCCO
279	X	X	N/A	Medical Arabization	KUWAIT
280	X	X	1681-5491	Medical Channel	PAKISTAN
281	X	√	1029-385X	Medical Forum	PAKISTAN
282	X	X	N/A	Medical Journal of Ahmed Maher Teaching Hospital [The]	EGYPT
283	X	X	0253-0759	Medical Journal of Basrah University [The]	IRAQ
284	√	X	0045-3803	Medical Journal of Cairo University [The]	EGYPT
285	X	X	1016-3360	Medical Journal of Islamic World Academy of Sciences	JORDAN
286	X	X	N/A	Medical Journal of Mashad University of Medical Sciences	ISLAMIC REPUBLIC OF IRAN
287	X	X	1726-7536	Medical Journal of Reproduction and Infertility	ISLAMIC REPUBLIC OF IRAN
288	X	X	1110-2039	Medical Journal of Teaching Hospitals and Institutes [The]	EGYPT
289	√	X	1016-1430	Medical Journal of the Islamic Republic of Iran	ISLAMIC REPUBLIC OF IRAN
290	X	X	N/A	Medical Journal of Tikrit University [The]	IRAQ
291	√	√	1011-7571	Medical Principles and Practice	KUWAIT
292	X	X	1023-5922	Medical Sciences Journal of Islamic Azad University	ISLAMIC REPUBLIC OF IRAN
293	X	X	0254-8534	Medical Spectrum [The]	PAKISTAN
294	X	X	1813-9876	Medicine Today	PAKISTAN
295	X	X	N/A	MEJO - Middle East Journal of Ophthalmolgy	SAUDI ARABIA
296	√	X	0544-0440	Middle East Journal of Anesthesiology	LEBANON
297	X	√	N/A<br>1729-6455	Middle East Journal of Emergency Medicine [The]	QATAR
298	X	X	N/A	Middle East Journal of Family Medicine	LEBANON
299	X	X	1110-2098	Minoufiya Medical Journal	EGYPT
300	X	X	1110-5437	MJFCT - Mansoura Journal of Forensic Medicine and Clinical Toxicology	EGYPT
301	X	X	0379-2617	Mother and Child	PAKISTAN
302	√	√	0027-8874	National Cancer Institute Journal	EGYPT
303	X	X	1687-0492	Neonatology	EGYPT

No	INDEXED IN MEDLINE	INDEXED IN EMBASE	ISSN	Journal Title	Country
304	X	√	1319-6138	Neurosciences	SAUDI ARABIA
305	√	X	1110-1946	New Egyptian Journal of Medicine [The]	EGYPT
306	X	X	1917-5562	New Iraqi Journal of Medicine [The]	IRAQ
307	X	X	1753-5001	Nursing and Midwifery Research	ISLAMIC REPUBLIC OF IRAN
308	X	X	N/A	Oman Medical Journal	OMAN
309	X	X	N/A	Omdurman Journal of Pharmaceutical Sciences	SUDAN
310	X	X	0030-9648	PAFMJ - Pakistan Armed Forces Medical Journal	PAKISTAN
311	X	X	0048-2706	Pakistan Heart Journal	PAKISTAN
312	X	X	N/A	Pakistan Jornal of Medical and Health Services	PAKISTAN
313	X	X	0300-8185	Pakistan Journal of Biochemistry	PAKISTAN
314	X	X	N/A	Pakistan Journal of Chest Medicine	PAKISTAN
315	X	X	1019-438X	Pakistan Journal of Clinical Psychology	PAKISTAN
316	X	X	N/A	Pakistan Journal of Community Medicine [The]	PAKISTAN
317	√	X	N/A	Pakistan Journal of Health	PAKISTAN
318	X	√	1682-024X	Pakistan Journal of Medical Sciences	PAKISTAN
319	X	X	N/A	Pakistan Journal of Neurology	PAKISTAN
320	X	X	N/A	Pakistan Journal of Obstetrics and Gynaecology	PAKISTAN
321	X	X	1608-134X	Pakistan Journal of Orthodontics, Pediatric and Community Dentistry	PAKISTAN
322	X	X	0257-4985	Pakistan Journal of Otolaryngology - Head and Neck Surgery	PAKISTAN
323	X	X	N/A	Pakistan Journal of Pathology	PAKISTAN
324	√	X	1011-601X	Pakistan Journal of Pharmaceutical Sciences	PAKISTAN
325	X	X	0255-7088	Pakistan Journal of Pharmacology	PAKISTAN
326	X	X	1819-270X	Pakistan Journal of Physiology	PAKISTAN
327	√	X	0030-9869	Pakistan Journal of Psychology	PAKISTAN
328	√	X	0030-9885	Pakistan Journal of Scientific and Industrial Research	PAKISTAN
329	X	X	0031-000X	Pakistan Medical Journal	PAKISTAN
330	X	X	0259-3661	Pakistan Ophthalmology	PAKISTAN

No	INDEXED IN MEDLINE	INDEXED IN EMBASE	ISSN	Journal Title	Country
331	X	X	1012-8700	Pakistan Oral and Dental Journal	PAKISTAN
332	√	X	0304-4904	Pakistan Pediatric Journal	PAKISTAN
333	X	√	1319-6995	Pan Arab Journal of Neurosurgery	SAUDI ARABIA
334	X	X	1607-4912	Pan Arab Journal of Orthopaedic and Trauma	EGYPT
335	X	X	N/A	Pan Arab Medical Journal	JORDAN
336	X	X	1735-1022	Pejouhandeh: Bimonthly Research Journal	ISLAMIC REPUBLIC OF IRAN
337	X	X	1016-1244	PJC - Pakistan Journal of Cardiology	PAKISTAN
338	√	X	0030-9842	PJMR - Pakistan Journal of Medical Research	PAKISTAN
339	X	X	0258-8552	PJS - Pakistan Journal of Surgery	PAKISTAN
340	X	X	N/A	PMJ - Palestinian Medical Journal	PALESTIN
341	√	X	0258-1914	Population Bulletin of ESCWA	IRAQ
342	X	X	N/A	Population Researches and Studies	EGYPT
343	√	X	N/A	Population Sciences	EGYPT
344	X	X	1110-1342	Population Studies	EGYPT
345	X	X	N/A	Proceedings	PAKISTAN
346	X	X	1024-8919	Professional Medical Journal - Quarterly [The]	PAKISTAN
347	X	√	0253-8253	Qatar Medical Journal	QATAR
348	X	X	1028-6918	Quarterly Journal of Fundamentals of Mental Health [The]	ISLAMIC REPUBLIC OF IRAN
349	X	X	N/A	Rehabilitation International	SAUDI ARABIA
350	X	X	1019-5335	Research Centre Bulletin	SAUDI ARABIA
351	X	X	N/A	Research Journal of Aleppo University - Medical Sciences Series	SYRIAN ARAB REPUBLIC
352	X	X	0330-7611	Revue Maghrebine de Pediatrie [La]	TUNISIA
353	X	X	N/A	Revue Maghrebine d'Endocrinologie - Diabete et de Reproduction [La]	TUNISIA
354	X	X	N/A	Revue Marocaine de Chirurgie Orthopedique et Traumatologique	MOROCCO
355	X	X	0251-0758	Revue Marocaine de Medecine et de Sante	MOROCCO
356	X	X	N/A	Revue Marocaine des Maladies de L'Enfant	MOROCCO
357	X	X	N/A	Risafa Medical Journal	IRAQ

No	INDEXED IN MEDLINE	INDEXED IN EMBASE	ISSN	Journal Title	Country
358	X	X	0303-5212	RMJ - Rawal Medical Journal	PAKISTAN
359	X	X	N/A	Salmand: Iranian Journal of Aging	ISLAMIC REPUBLIC OF IRAN
360	X	X	N/A	Sana'a University Medical Journal	YEMEN
361	X	X	1319-3965	Saudi Epidemiology Bulletin	SAUDI ARABIA
362	X	X	1018-077X	Saudi Heart Journal	SAUDI ARABIA
363	X	X	1319-6499	Saudi Journal of Disability and Rehabilitation	SAUDI ARABIA
364	X	X	1319-3767	Saudi Journal of Gastroenterology [The]	SAUDI ARABIA
365	X	X	1319-4534	Saudi Journal of Ophthalmology	SAUDI ARABIA
366	X	X	1319-8491	Saudi Journal of Oto-Rhino-Laryngology Head and Neck Surgery [The]	SAUDI ARABIA
367	X	X	1319-6308	Saudi Journal of Sports Medicine [The]	SAUDI ARABIA
368	√	√	0379-5284	Saudi Medical Journal	SAUDI ARABIA
369	X	X	1110-2381	Scientific Journal of Al-Azhar Medical Faculty [Girls] [The]	EGYPT
370	X	X	1027-1457	Scientific Journal of Forensic Medicine	ISLAMIC REPUBLIC OF IRAN
371	X	X	1560-652X	Scientific Journal of Kurdistan University of Medical Sciences	ISLAMIC REPUBLIC OF IRAN
372	X	X	1735-7586	Scientific Journal of School of Public Health and Institute of Public Health Research	ISLAMIC REPUBLIC OF IRAN
373	X	X	1110-5607	Scientific Medical Journal	EGYPT
374	X	X	N/A	Scientific Nursing Journal	IRAQ
375	X	X	1013-9052	SDJ - Saudi Dental Journal [The]	SAUDI ARABIA
376	X	X	1605-8941	Shaheed Beheshti Uneversity of Medical Sciences and Health Services	ISLAMIC REPUBLIC OF IRAN
377	X	X	N/A	Social Welfare Quarterly	ISLAMIC REPUBLIC OF IRAN
378	X	X	1110-7529	South Valley Medical Journal	EGYPT
379	X	√	1319-0164	SPJ - Saudi Pharmaceutical Journal	SAUDI ARABIA
380	X	X	1029-4066	SQUMJ - Sultan Qaboos University Medical Journal	OMAN
381	X	X	N/A	SST - Sante et Securite au Travail	TUNISIA
382	X	X	1735-4242	Strides in Development of Medical Education	ISLAMIC REPUBLIC OF IRAN
383	√	X	N/A	Sudan Journal of Medical Sciences	SUDAN
384	√	X	0491-4481	Sudan Medical Journal	SUDAN

No	INDEXED IN MEDLINE	INDEXED IN EMBASE	ISSN	Journal Title	Country
385	X	X	1585-5000	Sudan Medical Monitor	SUDAN
386	X	X	N/A	Sudanese Journal of Dermatology	SUDAN
387	X	X	N/A	Sudanese Journal of Public Health	SUDAN
388	X	X	1110-6999	Suez Canal University Medical Journal	EGYPT
389	X	X	N/A	Tabib Attifil Alarabi	LYBIAN ARAB JAMAHIRIYA
390	X	X	1735-0344	Tanaffos	ISLAMIC REPUBLIC OF IRAN
391	X	X	1110-1415	Tanta Medical Journal	EGYPT
392	X	X	1687-5788	Tanta Medical Sciences Journal	EGYPT
393	X	X	N/A	Tehran University Medical Journal [TUMJ]	ISLAMIC REPUBLIC OF IRAN
394	X	X	1728-5127	Toloo-e-Behdasht	ISLAMIC REPUBLIC OF IRAN
395	√	√	0041-4131	Tunisie Medicale [La]	TUNISIA
396	X	X	1606-8947	University of Aden Journal of Natural and Applied Sciences	YEMEN
397	√	X	1735-1308	Urology Journal	ISLAMIC REPUBLIC OF IRAN
398	X	X	1110-1423	Veterinary Medical Journal	EGYPT
399	X	√	1561-4921	Yakhteh Medical Journal	ISLAMIC REPUBLIC OF IRAN
400	X	X	N/A	Yemeni Journal for Medical Sciences [The]	YEMEN
401	X	X	N/A	YHMRJ - Yemeni Health and Medical Research Journal	YEMEN
402	X	X	N/A	YJMHR-Yemeni Journal of Medical and Health Research	YEMEN
403	X	X	N/A	YMJ - Yemen Medical Journal	YEMEN
404	X	X	N/A	Zagazig Journal of Forensic Medicine and Toxicology	EGYPT
405	X	X	1110-5089	Zagazig Journal of Pharmaceutical Sciences	EGYPT
406	X	X	1110-2322	Zagazig Medical Association Journal	EGYPT
407	X	X	1110-1431	Zagazig University Medical Journal	EGYPT
408	X	X	N/A	Zanco Journal for Medical Sciences	IRAQ
