# Supplementary material for: Index medicus for the Eastern Mediterranean region
Source: Emerg Themes Epidemiol. 2008 Sep 30;5:14. doi: 10.1186/1742-7622-5-14 (PMC2565659; doi:10.1186/1742-7622-5-14)
Supplement: Additional file 2 — Abstract in Arabic [file 1742-7622-5-14-S2.pdf]

أللغة العربية / Arabic

رؤية تحليلية

الكشاف الطبي لإقليم شرق المتوسط

المؤلف: الدكتور نجيب الشربجي

الملخص

تستعرض الدراسة مبررات وتاريخ الكشاف الطبي لإقليم شرق المتوسط لمنظمة الصحة العالمية و وضعه الراهن ، فالكشاف فريد من حيث التغطية الجغرافية للمجلات الصحية والطبية البيولوجية المحكَمة ويشتمل الكشاف على408 عناوين من 22 بلداً من بلدان الإقليم. ويعد تجميع ونشر الكشاف الطبي مع خدمة إيتاء الوثائق جزءاً لا يتجزأ من برنامج إدارة المعارف وتبادلها في المكتب الإقليمي للمنظمة. ونستعرض في هذه الورقة مؤشرات إحصائية للتعرُّف على مدى توزع المجلات والمقالات واللغات والمواضيع والمؤلفين إلى جانب مدى توافر ذلك في شكل إلكتروني وآخر مطبوع. ويساهم اثنان من بُلْدان الإقليم (وهما مصر وباكستان) في ما يزيد على 50% من المقالات في هذا الكشاف ، كما أن ما يقرب من 90% من المقالات منشورة باللغة الإنكليزية، وتمثل المقالات حول الوبائيات 8% من مجمل الكشاف ، وتتمتع 15% من المجلات بالتغطية في الكشاف الطبي على الخط المباشر MEDLINE، إلى جانب 7% منها مغطى في قاعدة معطيات المستخلصات الطبية EMBASE. وستتناول التطورات المستقبلية المرتقبة للكشاف الطبي بما في ذلك تغطية المزيد من المجلات مع إضافة أنماط أخرى من المطبوعات الصحية والطبية البيولوجية والتي تشمل التقارير ورالرسائل الجامعية والكتب والبحوث الجارية. وفي المقالة استعراض للدروس المستفادة ومناقشة لها.
