# Supplementary material for: Index medicus for the Eastern Mediterranean region
Source: Emerg Themes Epidemiol. 2008 Sep 30;5:14. doi: 10.1186/1742-7622-5-14 (PMC2565659; doi:10.1186/1742-7622-5-14)
Supplement: Additional file 3 — Abstract in Chinese – Simplified characters [file 1742-7622-5-14-S3.pdf]

Simplified Chinese / 简体中文

分析透视

**东地中海区域医学索引**

作者：Najeeb Al-Shorbaji

摘要：

本文介绍世界卫生组织东地中海区域医学索引的理念、历史及现况。该索引的独特之处在于其地理覆盖面涵盖了来自该区域的 22 个国家的（合共 408 份）经过同行评审的卫生与生物医学期刊。编纂和出版该索引，以及提供文件递送服务是世界卫生组织东地中海区域办事处知识管理及共享计划的一个主干部份。本文提供文献计量学指标以说明期刊、文章、语言、主题与作者的分布以及期刊有没有以印刷与电子形式出版。索引里有超过 50%的文章是投稿自本区域中的两个国家（埃及及巴基斯坦）的。大约 90%的文章是以英文出版的。流行病学的文章占索引全部文章的 8%。索引里的期刊有 15%亦被编入 MEDLINE 索引里，而有 7%被编入 EMBASE 里。索引的未来发展包括涵盖更多的期刊和加入其他类型的卫生及生物医学文献，包括报告、学位论文、书籍及进行中的研究。本文亦讨论了遇到的挑战及学习到的教训。

（中文摘要由冯俊熙翻译）
