# Supplementary material for: Index medicus for the Eastern Mediterranean region
Source: Emerg Themes Epidemiol. 2008 Sep 30;5:14. doi: 10.1186/1742-7622-5-14 (PMC2565659; doi:10.1186/1742-7622-5-14)
Supplement: Additional file 4 — Abstract in Chinese – Traditional characters [file 1742-7622-5-14-S4.pdf]

Traditional Chinese / 繁體中文

分析透視

東地中海區域醫學索引

作者：Najeeb Al-Shorbaji

摘要：

本文介紹世界衛生組織東地中海區域醫學索引的理念、歷史及現況。該索引的獨特之處在於其地理覆蓋面涵蓋了來自該區域的 22 個國家的（合共 408 份）經過同行評審的衛生與生物醫學期刊。編纂和出版該索引，以及提供文件遞送服務是世界衛生組織東地中海區域辦事處知識管理及共享計劃的一個主幹部份。本文提供文獻計量學指標以說明期刊、文章、語言、主題與作者的分佈以及期刊有沒有以印刷與電子形式出版。索引裡有超過 50%的文章是投稿自本區域中的兩個國家（埃及及巴基斯坦）的。大約 90%的文章是以英文出版的。流行病學的文章佔索引全部文章的 8%。索引裡的期刊有 15%亦被編入 MEDLINE 索引裡，而有 7%被編入 EMBASE 裡。索引的未來發展包括涵蓋更多的期刊和加入其他類型的衛生及生物醫學文獻，包括報告、學位論文、書籍及進行中的研究。本文亦討論了遇到的挑戰及學習到的教訓。

（中文摘要由馮儁熙翻譯）
