# Supplementary material for: Index medicus for the Eastern Mediterranean region
Source: Emerg Themes Epidemiol. 2008 Sep 30;5:14. doi: 10.1186/1742-7622-5-14 (PMC2565659; doi:10.1186/1742-7622-5-14)
Supplement: Additional file 5 — Abstract in French [file 1742-7622-5-14-S5.pdf]

French / Français

Perspectives Analytiques

Index Medicus pour la Région Est-Méditerranéenne

Auteur: Dr. Najeeb M. A. Al-Shorbaji

Résumé

Cette étude décrit le fonctionnement, l'histoire et l'état actuel de l'Index Medicus pour la Région Est-Méditerranéenne de l'Organisation Mondiale de la Santé. L'Index est unique du fait qu'il combine la couverture géographique des revues à comité de lecture de santé et biomédecine (408 titres) des 22 pays de la Région. La compilation et la publication de l'Index en juxtaposition avec un service de distribution des documents font partie intégrale du programme de gestion et de diffusion du savoir du bureau régional de L'OMS. Dans cet article sont présentés les indicateurs bibliométriques afin de montrer la distribution des revues, articles, langues, sujets et auteurs ainsi que la disponibilité en format papier et électronique. Deux pays dans la Région (l'Egypte et le Pakistan) contribuent pour plus de 50% des articles de l'Index. Environ 90% des articles sont publiés en anglais. Les articles d'Epidémiologie représentent 8% du contenu de l'Index. 15% des revues de l'Index sont aussi indexées sur MEDLINE, tandis que 7% sont indexées sur EMBASE. Les futurs développements de l'Index incluront la

couverture de plus de revues et l’ajout d’autres types de littérature de santé et biomédecine comme par exemple des rapports, des thèses, des livres et de la recherche actuelle. Les défis et leçons apprises sont discutés.

(Traduit en français par Annick Bórquez)
