# Supplementary material for: Index medicus for the Eastern Mediterranean region
Source: Emerg Themes Epidemiol. 2008 Sep 30;5:14. doi: 10.1186/1742-7622-5-14 (PMC2565659; doi:10.1186/1742-7622-5-14)
Supplement: Additional file 6 — Abstract in Spanish [file 1742-7622-5-14-S6.pdf]

Spanish / Español

Perspectiva Analítica

Index Medicus para la Región Este-Mediterránea

Autor: Dr. Najeeb M. A. Al-Shorbaji

Resumen

Este estudio describe el funcionamiento, la historia y el estado actual del Index Medicus para la Región Este-Mediterránea de la Organización Mundial de la Salud. El Index es único en combinar el abarcamiento geográfico de revistas revisadas por pares de salud y biomedicina (408 títulos) de los 22 países de la Región. Un objetivo integral del programa de gestión y difusión del conocimiento de la oficina de la OMS de la Región es compilar y publicar el Index en conjunto con un servicio integral de distribución de los documentos. En este artículo se presentan los indicadores bibliométricos usados para mostrar la distribución de revistas, artículos, idiomas, temas y autores así como la disponibilidad en formato impreso u electrónico. Dos países de la Región (Egipto y Pakistán) contribuyen con más de 50% de los artículos en el Index. Aproximadamente 90% de los artículos son publicados en inglés. Los artículos de epidemiología representan el 8% del contenido del Index. 15% de las revistas en el Index también están indexadas en MEDLINE, mientras que 7% están indexadas en EMBASE. Futuros desarrollos del Index incluirán abarcar más revistas y añadir otros tipos de literatura en salud y biomedicina incluyendo reportes, tesis, libros e investigación actual. Se discuten los retos y lecciones aprendidas.

(Traducido por Annick Bórquez)
